# Leveraging epigenetics to enhance the efficacy of immunotherapy

**DOI:** 10.1186/s13148-021-01100-x

**Published:** 2021-05-17

**Authors:** Jonathan D. Licht, Richard L. Bennett

**Affiliations:** Division of Hematology/Oncology, Department of Medicine, University of Florida Health Cancer Center, Cancer Genetics Research Complex, University of Florida, 2033 Mowry Road, Box 103633, Gainesville, FL 32610 USA

**Keywords:** Epigenetic therapy, Immune evasion, Immunotherapy, DNA methylation, Histone methyltransferase

## Abstract

**Background:**

Epigenetic mechanisms regulate chromatin accessibility patterns that govern interaction of transcription machinery with genes and their cis-regulatory elements. Mutations that affect epigenetic mechanisms are common in cancer. Because epigenetic modifications are reversible many anticancer strategies targeting these mechanisms are currently under development and in clinical trials.

**Main body:**

Here we review evidence suggesting that epigenetic therapeutics can deactivate immunosuppressive gene expression or reprogram tumor cells to activate antigen presentation mechanisms. In addition, the dysregulation of epigenetic mechanisms commonly observed in cancer may alter the immunogenicity of tumor cells and effectiveness of immunotherapies.

**Conclusions:**

Therapeutics targeting epigenetic mechanisms may be helpful to counter immune evasion and improve the effectiveness of immunotherapies.

## Background

### Epigenetic mechanisms in cancer

**Fig. 1:**
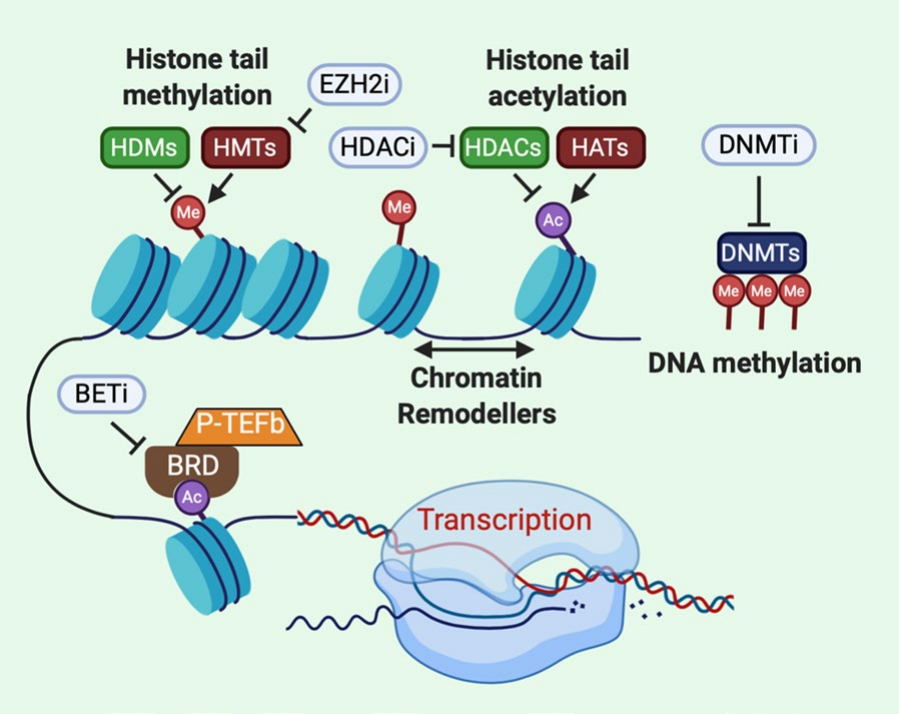
Epigenetic regulation of chromatin accessibility and gene expression. Nucleosomes (blue cylinders) are formed by DNA wrapped around a histone octamer which allows DNA to be condensed into chromatin and finally chromosomes. Epigenetic mechanisms dynamically tune chromatin accessibility especially at cis regulatory elements of gene expression. Post-translational modification of N- and C-terminal histone “tails” regulate nucleosome stability, chromatin compaction and serve as docking sites for proteins that recognize epigenetic marks such as bromodomain proteins (BRD). Histone methyltransferases (HMTs) catalyze the transfer of a methyl group (Me) onto histone tails from donor S-adenyl methionine while this mark is removed by histone demethylases (HDM). Similarly, histone acetyltransferases (HATs) transfer an acetyl group (Ac) from acetyl coenzyme A to histone lysine residues which weakens histone interaction with DNA to increase chromatin accessibility. Histone deacetylases (HDACs) remove the acetyl mark from histones, decreasing chromatin accessibility and subduing gene expression. Chromatin remodelers such as SWI/SNF mobilize and reposition nucleosomes. DNA methyltransferases (DNMT) methylate CpG islands near transcription start sites that inhibit gene expression by impeding transcription factor binding to DNA. Epigenetic inhibitors (white ovals) have been developed to potentially restore a normal cellular epigenetic state to tumor cells. EZH2i, such as tazemetostat, specifically inhibit the gene suppressive methylation of histone H3 by PRC2. HDACi such as entinostat or vorinostat inhibit histone deacetylation to reactivate gene expression. Inhibitors of bromodomain and extra terminal domain proteins (BETi) such as JQ1 or BMS-986158 suppress aberrant gene expression driven by increased BRD activity in cancer cells. DNMT inhibitors (DNMTi) such as azacytidine promote DNA hypomethylation and reactivate expression of tumor suppressor genes

Heritable patterns of gene expression not due to DNA sequence variation are maintained and regulated by epigenetic mechanisms. These mechanisms allow genetically identical cells to have distinct gene expression patterns that govern specialization of cellular identity. Patterns of DNA methylation, histone post-translational modifications or nucleosome positioning are epigenetic marks that reversibly store and transmit heritable information (Fig. 1). Writers of epigenetic marks catalyze DNA methylation or post-translational modifications of histones, such as methylation or acetylation of N-terminal histone “tails” extending from the nucleosome structure. These epigenetic marks may be recognized by protein complexes that either alter the chromatin architecture further or regulate enzymatic processes such as transcription factor binding and RNA polymerase processivity. Epigenetic marks may be removed by enzymes such as histone deacetylases or demethylases. In addition, chromatin remodelers can mobilize or exchange histones. Together these mechanisms maintain chromatin accessibility patterns that govern interaction of transcription machinery with genes and cis-regulatory regions. Mutations in genes that regulate the cellular epigenetic state are among the commonest class of mutations found in cancer [1]. These mutations result in reprogrammed gene expression that can directly contribute to cancer and cooperate with other genetic events such as mutations of oncogenes or tumor suppressors, affecting signal transduction and cell life/death pathways. Because epigenetic marks are reversible, many anticancer strategies targeting these mechanisms to rebalance the epigenome back to a more normal state are currently under development and in clinical trials. In addition, the widespread adoption of targeted immunotherapies for cancer has led to the need for a deeper understanding for how epigenetic mechanisms may allow tumor cells to escape immune surveillance.

### Patterns of DNA methylation regulate gene expression

Methylation of DNA in somatic cells occurs primarily on cytosines that precede a guanine (CpG) and is important for regulation of gene expression. Palindromic methylation patterns are maintained in the genome and transmitted through the germline. CpG dinucleotides are often concentrated within CpG-rich DNA “islands” located around transcription start sites (TSSs) [2]. Hypermethylation of CpG islands may repress expression of nearby genes by inhibiting transcription factor binding and recruiting methyl-CpG binding proteins which in turn affect repressive histone modifying enzymes [3]. Global and local gene methylation pattern changes are frequently found in cancer and atypical methylation patterns have been used to differentiate tumor subtypes [4]. Loss of repeat region methylation is often observed in tumors, and hypomethylation of DNA regions may promote the expression of proto-oncogenes such as observed for ERBB2 and RAS [5]. In addition, hypermethylation of specific CpG-rich regions may silence expression of tumor suppressors such as observed for Rb and p16 [6, 7].

The DNA methyltransferases (DNMT) family of enzymes is responsible for establishing and maintaining patterns of DNA methylation. DNMT3A and DNMT3B establish de novo DNA methylation patterns that are maintained by DNMT1, which recognizes DNA methylation and directs daughter strand methylation after replication [8]. DNA demethylation is expedited by the TET enzymes which convert methyl cytosine into hydroxymethylcytosine, a base modification not recognized by DNMT1 [9]. Mutations of DNMT3A or TET enzymes that cause loss of function are often observed in cancer, dysregulating patterns of DNA methylation.

### Histone acetylation regulates chromatin accessibility

Control of lysine acetylation in the histone tail region is important for regulation of chromatin structure, transcription and DNA repair. This highly dynamic modification is regulated by histone lysine acetyltransferases (HATs) and histone deacetylases (HDACs). HATs transfer the acetyl group from acetyl-coenzyme A to the amino group of a lysine in the histone, thereby neutralizing the lysine’s positive charge and weakening the interaction of histone with DNA. In general, histone acetylation causes a more relaxed and accessible chromatin structure that favors binding of proteins such as transcription factors. Thus, acetylation of chromatin is generally associated with transcriptional activation while deacetylation is associated with gene repression [10]. HDACs play a key role in gene expression by removal of the activating histone acetylation and may also have other roles in the cell by controlling acetylation of non-histone and non-nuclear proteins. HDACs are often found overexpressed in cancer where they may silence tumor suppressor genes. In addition, HDACs may be aberrantly recruited to target genes by overexpressed transcription factors or chimeric transcription factors created by chromosomal fusions [10].

### Bromodomain-containing proteins recognize acetylated histone

The bromodomain and extra-terminal (BET) protein family that includes BRD2, BRD3 and BRD4 is one of the best characterized families of epigenetic reader proteins in cancer [11, 12]. BET proteins share a conserved structural element consisting of two bromodomains that recognize acetylated lysine on the N-terminal tails of histones H3 and H4. In addition, BRD4 associates with the positive transcription elongation factor (P-TEFb) protein that promotes transcription elongation at paused sites by activating RNA polymerase II [13]. BRD4 is frequently located at transcription start sites, enhancer and super-enhancer regions. Significantly, BRD4 has been reported to promote expression of many transcription factors with roles in cancer development and progression such as Myc. In addition, BRD4 has been reported to recruit the histone methyltransferase NSD2 to the estrogen receptor alpha (ERα) gene to increase ER expression in breast cancer cells [14]. Increased BRD4 expression is associated with poor prognosis in melanoma and hepatocellular carcinoma. In addition, the majority of patients that develop midline carcinoma have chromosomal rearrangements that create a fusion protein between BRD3 or BRD4 and the NUT protein [13]. The BRD-Nut fusion maintains cancer cells in an undifferentiated state of self-renewal by recruiting HATs and sequestering cofactors normally associated with activated, acetylated chromatin away from normal gene targets, leading to reduced expression of differentiation-associated genes. Treatment of BRD4-NUT expressing cell lines with a small molecular inhibitor of BET domain acetyl-lysine binding led to a reactivation of gene expression and induction of differentiation [13]. This finding stimulated the ongoing development of pharmacological approaches to inhibit BET domain proteins.

### Histone methylation states regulate transcription

N-terminal and C-terminal tails of histones that extend beyond the nucleosome core are subject to post-translational modifications that influence downstream biological processes such as transcription, replication and chromosomal stability. The basic amino acids lysine, arginine and histidine present in histone tails can serve as a target of methylation. Reversible methylation of histones is orchestrated by histone methyltransferases (HMTs), while histone demethylases (HDMs) remove these marks (Fig. 1). The enzymatic activity of almost all lysine methyltransferases resides in the Su(var)3–9, enhancer-of-zeste and trithorax (SET) catalytic domain. The prime exception being the lysine methyltransferase DOT1L1 which has a unique enzymatic domain. Arginine methyl transferases contain a conserved S-adenosyl methionine (SAM)-binding catalytic core of about 350 amino acids [15]. These enzymatic domains have pockets that bind SAM to be used as a donor co-factor for the transfer of methyl groups to substrates. Histone lysine residues can be modified to mono-, di- or tri-methylated forms (me1, me2 or me3) and histidine can be mono-methylated, but this modification is rare. Arginine may be mono- or di-methylated, and di-methylation may be either symmetrical, meaning that methyl groups are added to both nitrogen atoms in the side chain or asymmetrical in which two methyl groups are added to only one of the side chain nitrogen atoms. Demethylation of histones is accomplished by two main classes of HDMs: the flavin adenine dinucleotide (FAD)-dependent amine oxidases and the Fe(II)/2-oxoglutarate (2-OG)-dependent Jumonji C domain family [15]. Patterns of histone methylation state can change based on cell type, tissue type or cell cycle phase. The reversible nature of histone methylation is important for the response to factors such as DNA damage, mitogen signaling and environmental stress because the balance between the methylated and demethylated states of histones at specific lysine residues can regulate transcriptional activity. For instance, lysine methylation of histone H3 at amino acid residue 4 (H3K4), 36 (H3K36) and 79 (H3K79) are associated with a gene activation state while methylation at lysine 9 (H3K9), 20 (H3K20) and 27 (H3K27) is associated with suppression of gene expression [16]. Loss or gain of HMT or HDM activity can result from missense mutation, deletion, amplification or chromosomal rearrangement affecting the genes encoding these enzymes. Dysfunctional histone methylation and the resultant aberrant gene expression have often been linked to a range of malignancies and clinical outcome.

Enhancer-of-zeste homolog 2 (EZH2) is the catalytic component of the polycomb repressive complex 2 (PRC2) that trimethylates histone H3 at lysine 27 (H3K27) which is associated with chromatin compaction and transcriptional repression [17]. EZH2-mediated H3K27 methylation is an important regulator in several cellular pathways including cell cycle regulation, X-chromosome inactivation and metastasis. EZH2 expression is generally found to be increased in metastatic tumors compared to normal tissues or primary tumor specimens. Increased EZH2 promotes cancer cell growth and an epithelial-mesenchymal transition. Heterozygous activating mutations of EZH2 are found in germinal center-type diffuse large B cell lymphoma and more rarely in thyroid cancer and malignant melanoma [18]. In lymphoma, gain of function EZH2 mutations repress expression of tumor suppressor and late B cell genes, thus locking the B cell in a state of continuous proliferation at the germinal center stage of differentiation. In addition, loss of function and deletion of EZH2 is found in MDS and AML and is associated with global decreases in the repressive H3K27me3 mark which activates expression of oncogenes [19]. Thus, EZH2 can have either an oncogenic or tumor suppressor function, depending on the cellular context.

## Main text

### Epigenetic therapies may synergize with immunotherapy

**Fig. 2:**
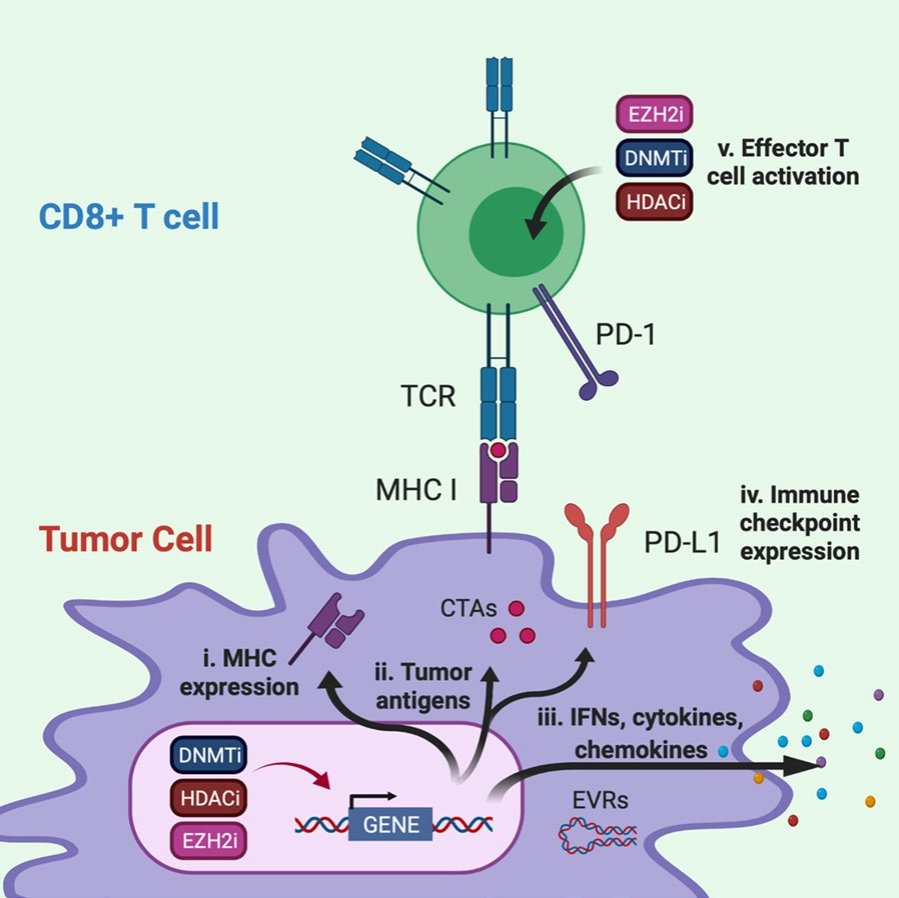
Leveraging inhibition of epigenetic mechanisms to improve immunotherapy. DNA methyltransferase inhibitors (DNMTi), histone deacetylase inhibitors (HDACi) and an inhibitor of histone methylation on histone H3 at lysine 27 (EZH2i) activate immunomodulatory mechanisms that may improve immunotherapy by: (i) increasing gene expression and activation of antigen presentation mechanisms; (ii) increasing gene expression of tumor-associated antigens such as cancer testis antigens (CTAs) MAGE and NY-ESO-1; (iii) upregulating inflammatory genes and pathways that rebalance the secretion of interferons (IFNs), cytokine and chemokines from tumor cells including the expression of normally silent endogenous retroviruses RNAs (EVRs) that activate the interferon response; (iv) upregulating targets of immune checkpoint blockade such as PD-1/PD-1L on both tumors and lymphocytes; and (v) activating the effector T cell population by promoting differentiation of naïve T cells to cytotoxic T cells and inhibiting T cell exhaustion mechanisms

A recurring phenotype of cancer cells is aberrant epigenetic mechanisms causing the downregulation of genes involved in the processing or presentation of tumor antigens, leading to immune evasion [20–22]. Many pharmacological agents have been developed that inhibit epigenetic mechanisms and reprogram tumor cell-specific patterns of DNA methylation or post-translational histone modifications. Furthermore, the complex interplay between immune, cancer and stromal cells is important for antitumor immunity. The therapeutic potential of combining epigenetic therapies with immunotherapy was first indicated by reports demonstrating that immune- or inflammatory-related gene signatures were increased upon inhibition of epigenetic mechanisms [23–26]. Furthermore, early clinical studies of combination therapies found that non-small cell lung cancer (NSCLC) patients treated with either DNMT or HDAC inhibitors achieved durable treatment responses when subsequently administered PD1/PD-L1 immune checkpoint inhibitors [27, 28]. These studies and others suggest that epigenetic inhibitors may increase the efficacy of immunotherapy by: (1) enhancing antigenicity and presentation of tumor-associated antigens, (2) reprogramming the tumor microenvironment to counteract immunosuppressive mechanisms and (3) reversing cytotoxic T cell exhaustion (Fig. 2). As our knowledge of how epigenetic mechanisms govern tumor antigen presentation and immune cell function improves, strategies that take advantage of these mechanisms will be important to devise rational combinatorial approaches that bolster response to immunotherapies.

### DNA hypomethylation agents synergize with immunotherapy

Inhibitors of DNA methyltransferases (DNMTi) were developed in the 1970s to target aberrant methylation patterns in cancer cells. DNMTi such as 5-azacytidine, decitabine and guadecitabine cause global hypomethylation, and their use as anti-cancer agents has been approved for use in patients with myelodysplastic syndrome or certain leukemias to reactivate tumor suppressor genes. These azanucleosides substitute nitrogen for carbon at the C-5 position of the pyrimidine ring and when incorporated into DNA irreversibly bind DNMT1 resulting in DNMT1 degradation and decreased DNA methylation [29]. The resulting loss of DNA methylation favors the re-expression of aberrantly silenced proteins, including tumor suppressor genes, cancer-associated antigens and components of the antigen presentation machinery.

Emerging evidence suggests that DNMTi hypomethylating agents have potential to improve immunogenicity and immune recognition of cancer cells. Numerous preclinical studies demonstrate that hypomethylating agents significantly increase expression of immunomodulatory pathway genes and tumor antigen presentation mechanisms in a variety of human epithelial cancer cell lines [23, 30]. Antigen presentation mechanisms found upregulated include the type I interferon response pathway upstream of antigen presentation, immune proteasome subunits and endoplasmic reticulum transporters involved in antigen processing prior to presentation and MHC class I genes required for antigen presentation to cytotoxic T cells [23, 24, 31]. For instance, in NSCLC cell lines, 5-azacytidine treatment upregulates the JAK/STAT pathway stimulating the expression of genes involved in antigen presentation and increasing expression of PD-L1, a key ligand-mediator of immune tolerance [27, 32]. DNMTi were also reported to activate the expression of endogenous retroviral double-stranded RNAs (EVRs) that are normally hypermethylated and transcriptionally silent, leading to the induction of a type I interferon response and the activation of MHC I expression [26, 33, 34]. Recent studies indicate that expression of EVRs in renal cell carcinoma, breast, colon and head and neck squamous cell tumors correlated with increased immune infiltration, checkpoint pathway upregulation and higher CD8 + T cell infiltration [35, 36]. Significantly, high EVR expression in tumors correlated with improved response to anti-CTLA4 in melanoma patients and PD-1/PD-L1 blockade in renal cell carcinoma patients [26, 35]. Additionally, DNMTi can upregulate expression of cancer testis antigens (CTAs) which are promising immunotherapy targets expressed in early embryonic and germ cells but normally silenced in mature somatic cells by promoter CpG island DNA methylation [37, 38]. CTAs, such as NY-ESO-1 or MAGE-a, are expressed in a variety of tumor cell types and increased expression is associated with advanced or metastatic disease stage and poor prognosis [39–41].

DNMTi such as decitabine not only affect tumor cells but may also have direct effects on antigen-specific CD8 + T cells. Genome-wide de novo DNA-methylation programs persist in CD8 + T cells after PD-1 immune checkpoint blockade therapy and this restricts the durability of therapy by promoting terminal differentiation of exhausted T cells. Studies in tumor-bearing mice indicate that prior treatment with decitabine followed by administration of anti-PD-L1 prevents the acquisition of exhaustion-associated methylation programs, and T cells retain a greater potential for expansion after immune checkpoint blockade [42]. Furthermore, in a murine ovarian cancer model the efficacy of anti-CTLA-4 was potentiated by combination with decitabine which increased differentiation of naive T cells into effector T cells. This in turn prolonged cytotoxic lymphocyte responses as well as mouse survival [43].

Early-stage clinical trials testing the second-generation DNMTi guadecitabine with ipilimumab (anti-CTLA-4) in metastatic melanoma patients showed promising tumor immunomodulatory and clinical activities [44]. Results indicate that combination therapies utilizing hypomethylating agents promoted upregulation of HLA class I molecules and IFN gamma signaling pathways as well as increased tumor infiltration by CD8 + T cells, hallmarks for sensitivity to immunotherapy [44]. In a phase I clinical trial of relapsed epithelial ovarian cancer, administration of decitabine significantly increased NY-ESO-1 expression, and T cell responses were increased after treatment with the NY-ESO-1 epitope, leading to a favorable response in 60% of patients [45]. Taken together these studies have guided the design and initiation of additional clinical trials that test DNMTi hypomethylating agents in combination with immunotherapies (Table 1).

**Table 1:** Clinical trials evaluating combination of epigenetic inhibitors and immunotherapies

Epigenetic therapy	Immunotherapy	Cancer type	Phase, Trial ID
*Histone deacetylase inhibitors (target)*			
CXD101 (Pan HDAC)	Nivolumab (PD-1)	Colorectal cancer	I/II, NCT03993626
Domatinostat (HDAC1,2,3)	Avelumab (PD-L1)	GI cancer	II, NCT03812796
Entinostat (HDAC1,2,3)	Pembrolizumab (PD-1)	Bladder cancer	II, NCT03978624
		Melanoma	II, NCT03765229
		MDS	I, NCT02936752
		Metastatic uveal melanoma	II, NCT02697630
	Atezolizumab (PD-L1)	Breast cancer	I/II, NCT03280563
	Nivolumab (PD-1)	Cholangiocarcinoma, pancreatic adenocarcinoma	II, NCT03250273
	Aldesleukin (IL-2)	Renal cell carcinoma	I/II, NCT01038778
	Nivolumab (PD-1), Ipilimumab (CTLA-4)	Breast cancer	I, NCT02453620
Mocetinostat (Pan HDAC)	Durvalumab (PD-L1)	NSCLC	I/II, NCT02805660
Tinostamustine (Pan HDAC)	Nivolumab (PD-1)	Melanoma	I, NCT03903458
Vorinostat (Pan HDAC)	Pembrolizumab (PD-1)	Lymphomas	I, NCT03150329
		Renal cell carcinoma	I, NCT02619253
		NSCLC	I/II, NCT02638090
		Head and neck	I/II, NCT02538510
*DNA methyltransferase inhibitors*			
Azacytidine	Avelumab (PD-L1)	DLBCL	III, NCT02951156
	Alemtuzumab (CD52)	Myeloid malignancies	II, NCT02497404
	Pembrolizumab (PD-1)	AML	II, NCT02845297
		AML	II, NCT03769532
		Pancreatic cancer	II, NCT03264404
		MDS	II, NCT03094637
Oral azacytidine (CC-486)	Pembrolizumab (PD-1)	Ovarian cancer	II, NCT02900560
		NSCLC	II, NCT02546986
		Melanoma	II, NCT02816021
Decitabine	Pembrolizumab (PD-1)	T cell lymphomas	II, NCT03240211
		Lymphomas	I, NCT03445858
		AML	I, NCT03969446
		Breast cancer	II, NCT02957968
	Anti-PD-1 antibody	Solid tumors	I/II, NCT02961101
	Dendritic cell vaccine (NY-ESO-1, MAGE-A1 MAGE-A3)	Pediatric brain tumors	I/II, NCT02332889
Guadecitabine	Atezolizumab (PD-L1)	Urothelial carcinoma	II, NCT03179943
	Durvalumab (PD-L1)	Liver, pancreatic, bile duct, gallbladder	I, NCT03257761
	GVAX (Cell vaccine)	Colon cancer	I, NCT01966289
	Ipilimumab (CTLA-4)	Melanoma	I, NCT02608437
	Pembrolizumab (PD-1)	Ovarian	II, NCT02901899
		Prostate, NSCLC	I, NCT02998567
*Histone modifications (target)*			
Tazemetostat (EZH2)	Pembrolizumab (PD-1)	Bladder cancer	I/II, NCT03854474
CPI-1205 (EZH2)	Ipilimumab (CTLA-4)	Solid tumors	I/II, NCT03525795
BMS-986158 (BRD2/3/4, BRDT)	Nivolumab (PD-1)	Advanced tumors	I/II, NCT02419417
*Multiple combinations*			
Azacytidine, entinostat	Nivolumab	NSCLC	II, NCT01928576
Azacytidine, venetoclax (Bcl-2)	Pembrolizumab	AML	II, NCT04284787
Azacytidine, epacadostat (IDO-1)	Pembrolizumab	Metastatic solid tumors	I/II, NCT02959437
Mocetinostat, guadecitabine	Pembrolizumab	Lung cancer	I, NCT03220477
Vorinostat, temozolomide	Pembrolizumab	Glioblastoma	I, NCT03426891
Vorinostat, tamoxifen	Pembrolizumab	Breast cancer	II, NCT04190056 II, NCT02395627
Multiple agents	Multiple agents	Breast, prostrate, pancreas, AML	I, NCT03878524
Azacytidine, romidepsin (Pan HDAC)	Pembrolizumab	Colorectal cancer	I, NCT02512172
Azacytidine	Tremelimumab (CTLA-4) Durvalumab (PD-L1)	Head and neck cancer	I/II, NCT03019003
Decitabine, tetrahydrouridine	Pembrolizumab	NSCLC	I/II, NCT03233724

### Histone deacetylase inhibitors promote an immunotherapy favorable microenvironment

Acetylation of lysine on histone tails is commonly observed at promoter and enhancer gene regulatory regions of actively transcribed genes. In cancer cells, de-acetylation of histone lysine residues is commonly associated with hypermethylated and silenced genes. Histone deacetylase inhibitors (HDACi) target these regions to reactivate gene expression. Several HDACi, such as belinostat, panobinostat, romidepsin, vorinostat have received FDA approval for treatment of solid tumors and hematological malignancies and these are reviewed elsewhere [4, 46, 47]. HDACi have been demonstrated to influence tumor immunogenicity and the functional activity of specific immune cells. For instance, treatment of carcinoma cells with HDACi increased the expression of antigen processing enzymes and increased MHC class I expression on the surface of tumor cells [48].

Studies also suggest that HDACi may be useful for reprogramming the tumor microenvironment to deactivate immunosuppressive cells and increase cytotoxic T cell trafficking to the tumor. Cytokines and chemokines from tumor cells such as IL-6, IL-10, TGF-beta, VEGF and CCL2 promote exclusion of cytotoxic T cells from the tumor microenvironment and recruit immunosuppressive cells such as regulatory T cells, myeloid-derived suppressor cells, tumor-associated macrophages, and tolerogenic dendritic cells. Recent reports indicate that HDAC11 and HDAC6 interact with each other in the cytoplasm and nuclei of antigen-presenting cells to coordinate regulation of the IL-10 promoter. Interestingly, HDAC11 was shown to repress IL-10 gene transcriptional activity in APCs while HDAC6 promoted the expression of IL-10 as a transcriptional activator [49]. Overexpression of HDAC11 inhibited IL-10 expression and induced inflammatory APCs that were able to prime naive T cells and restore the responsiveness of tolerant CD4 + T cells [50]. In a mouse melanoma model, pretreatment with HDAC6 inhibitor Nexturastat A followed by administration of anti-PD-1 blocking antibodies significantly decreased tumor growth by causing increased infiltration of CD8 + T cells and natural killers cells as well as a reduction of pro-tumoral M2 macrophages in the tumor microenvironment [51]. Furthermore, cotreatment of syngeneic mouse tumor models with the HDACi entinostat and 5-azacytidine markedly improved the response to both anti–PD-1 and anti–CTLA-4 checkpoint inhibitor antibodies, curing more than 80% of the tumor-bearing mice [52]. Functional studies revealed that rather than alter the level of CD8 + T cell infiltration or antigen presentation mechanisms, entinostat decreased the viability of the myeloid-derived suppressor cell population [52]. Overall these studies suggest that selective HDACi could be used as immunological priming agents to sensitize immunologically "cold" tumors and subsequently improve immune checkpoint blockade therapies.

The promise of combination therapy that includes immune checkpoint blockade and HDACi has led to the recent initiation of several clinical trials. In a phase I study of patients with immune checkpoint inhibitor-resistant metastatic NSCLC, the combination of the anti-PD-1 antibody pembrolizumab and HDACi vorinostat resulted in one confirmed partial response and eight stable disease responses among 24 enrolled patients [53]. Moreover, preliminary results from the ENCORE-601 phase I/II trial evaluating the combination of pembrolizumab and HDACi entinostat demonstrate a favorable response in patients with colorectal cancer and immune checkpoint inhibitor-resistant melanoma or NSCLC [54–56].

### Inhibition of histone methylation promotes tumor immunogenicity

Numerous reports suggest that polycomb repressive complex 2 (PRC2)-mediated epigenetic silencing is a key mechanism facilitating immune evasion. For instance, PRC2 suppresses tumor antigen presentation mechanisms by transcriptionally repressing MHC class I antigen presentation pathway genes [57]. Treatment of cancer cell lines with a pharmacological EZH2 inhibitor (EZH2i) promoted antigen-specific T cell killing in vitro, and disruption of EZH2 in mouse models leads to re-establishment of a T cell-mediated anti-tumor response [57]. Furthermore, EZH2i can promote tumor immunogenicity by reactivating the expression of normally silent endogenous retroviruses [34]. EZH2 inhibition was also observed to promote IFN signaling and production of proinflammatory cytokines such as CXCL9 and CXCL10. Also, EZH2 has been reported to be crucial for immune cell differentiation. Targeted disruption of EZH2 in regulatory T cells enhances the anti-tumor immune response in mouse models [58, 59]. EZH2 deficiency in regulatory T cells not only reduces the frequency of these immune suppressive cells in tumors but also alters their function converting them from immunosuppressive to pro-inflammatory cytokine producers in tumors [59]. In a mouse model, inhibition of EZH2 in T cells increases the effectiveness of anti-CTLA-4 therapy [58]. Several EZH2i such as tazemetostat and CPI-1205 are currently in clinical trials as single agent or combination therapies for a variety of cancer types. For instance, the combination of tazemetostat and pembrolizumab is currently being tested in clinical trials of patients with urothelial carcinoma while CPI-1205 is being tested in combination with ipilimumab in patients with advanced solid tumors previously treated with PD-1 or PD-L1 inhibitors.

### Targeting microRNAs to improve tumor immunogenicity

Among the diverse epigenetic mechanisms that regulate gene expression, microRNAs (miRNAs) representant an attractive target for future strategies to augment immunotherapy. Non-protein-coding RNAs, including long noncoding RNAs (> 200 nucleotides) and small noncoding RNAs such as enhancer RNAs and miRNAs, can regulate transcription directly or at the post-transcription level to provide another layer of epigenetic control for gene expression. MiRNAs are a group of short (~ 22 nt), evolutionally conserved, single-stranded noncoding RNA molecules that prevent expression of specific target genes through sequence-specific RNA–RNA interactions with the 3′-untranslated regions of target mRNA. MiRNA-mediated gene silencing occurs by mRNA cleavage and degradation, or translational repression, depending on the degree of complementarity between the miRNA and the targeted mRNA [60, 61]. In addition, miRNAs may function as ligands of Toll-like receptor (TLR) to trigger activation of downstream signaling pathways [62]. Currently there are about 2500 curated miRNAs, and it has been estimated that almost 2/3rds of protein-encoding genes are regulated by miRNAs [63]. Compelling evidence demonstrates that miRNA expression is dysregulated in human cancer through various mechanisms, including amplification or deletion of miRNA genes, abnormal transcriptional control of miRNAs, dysregulated epigenetic changes and defects in the miRNA biogenesis machinery [64, 65]. The regulatory roles of miRNAs in metabolic and cellular pathways, especially those controlling cell proliferation, differentiation, apoptosis, and survival, are crucial to tumor initiation and progression [66]. Thus, miRNAs may function as either oncogenes or tumor suppressors depending on specific contexts.

MiRNAs are important to regulate immune cell function, differentiation and interaction with the tumor microenvironment, and these functions have been subject of several recent reviews [67, 68]. For instance, miRNAs such as miR-27a have been reported to alter tumor antigen presentation and colorectal cancer tumors with high miR-27a had reduced T cell infiltration. Increased miR-27a in colorectal cancer was associated with distant metastasis and poor prognosis [69]. In the tumor microenvironment, miRNAs, such as miR-101 and miR-222, regulate the interaction of cancer-associated fibroblasts with tumor cells [70, 71]. MiRNAs have also been reported to regulate immune checkpoints. For instance, miR-34 binds directly to the 3' untranslated region of PDL1 in non-small cell lung cancer cell lines to downregulate PDL-1 expression, resulting in enhanced T cell response [72, 73]. Thus, miRNAs may be useful biomarkers in some contexts to predict response to immunotherapies [68].

Strategies to target miRNAs in cancer are beginning to be developed and tested. Aberrant miRNA expression in cancer may be targeted depending on the cellular context by either restoring miRNA expression that has been lost in cells or inhibiting upregulated miRNAs. Multiple groups are pursing strategies to deliver miRNAs to tumors such as lipid encapsulated miRNA mimics or blockers as potential cancer therapeutics. For instance, a liposomal miR-34a mimic (MRX34) was recently evaluated in patients with advanced solid tumors refractory to all standard treatments [74]. MRX34 treatment with dexamethasone premedication demonstrated some clinical activity, but the trial was closed prematurely due to serious immune-mediated toxicities [74]. Furthermore, in a phase 1 clinical trial, “minicells” loaded with miR-16 and targeted to EGFR were assessed as having an acceptable safety profile and signs of clinical activity in mesothelioma patients [75]. Another group has evaluated an oligonucleotide inhibitor of miR-155, cobomarsen, in DLBCL cell lines and xenograft mouse models [76]. Cobomarsen reduced tumor volume, triggered apoptosis and derepressed direct miR-155 target genes [76]. Although studies such as these are early, they provide proof-of-concept for miRNA-based cancer therapy. As our understanding for how miRNAs may regulate the immune system and response to immunotherapy, a future therapeutic approach may be to integrate these strategies.

## Conclusions

Recently, an increasing number of pharmacological agents targeting epigenetic mechanisms have shown promise in clinical trials of cancer patients both as single agents and in combination with other therapies. Insights into epigenetic mechanisms, particularly as they relate to immune cell function and tumor antigen presentation, have informed recent rational strategies for targeting tumor cells and reversing acquired immunotherapy resistance. Epigenetic therapeutics can deactivate immunosuppressive gene expression or reprogram tumor cells to activate antigen presentation mechanisms. Importantly, additional work must be done to determine whether the benefit of integrating epigenetic therapy with immunotherapy may depend upon the type of cancer or some other specific context. Many epigenetic inhibitors have been reported to negatively impact the proliferation of T cells which could hinder the durability of immunotherapy that relies on a persistent T cell population. For instance, inhibition of the EZH2 and the PRC2 complex has been reported to undermine T cell function. EZH2 is essential for the development and maintenance of memory T cells that sustain effector T cell production and associated antitumor function [77]. EZH2-deficient CD8 + T cells were incapable of mediating tumor growth inhibition to the same degree as EZH2-sufficient cells when transferred into mice with preestablished B16-melanoma [77]. In addition, EZH2 has been reported to stimulate polyfunctional cytokine expression on T cells and promote T cell survival by BCL2 signaling [78]. Pharmacological inhibition of EZH2 or short hairpin RNA-mediated knockdown of EZH2 in T cells was reported to elicite poor antitumor immunity [78]. EZH2 + CD8 + T cell numbers and the percentage of EZH2 + CD8 + T cells in tissue samples of ovarian cancer patients were significantly associated with improved overall survival [78]. These studies suggest that more information regarding how epigenetic mechanisms regulate immunity is necessary to determine the circumstances a particular epigenetic therapy may be most beneficial for cancer patients and whether there is a therapeutic window to use such agents in combination with immunotherapy. Identification and validation of biomarkers may be required to determine which patients will derive the greatest benefit from combined epigenetic and immunotherapy. As our understanding of how epigenetic mechanisms may govern immunotherapy effectiveness grows, leveraging these discoveries to counter immune evasion will be valuable to shape future anti-cancer strategies and improve patient outcomes.

## Data Availability

Data sharing not applicable to this article as no datasets were generated or analyzed during the current study.

## Abbreviations

EZH2: Enhancer of zeste homolog 2
PRC2: Polycomb repressive complex 2
H3: Histone H3
DLBCL: Diffuse large B cell lymphoma
DNMT: DNA methyltransferase
HDAC: Histone deacetylase
CTA: Cancer testis antigen
EVR: Endogenous viral RNA
miRNA: MicroRNA
NSCLC: Non-small cell lung cancer
